# Principles of Human Physiology, with Their Chief Applications to Pathology, Hygiene, and Forensic Medicine

**Published:** 1845-04

**Authors:** 


### Principles of Human Physiology, with their chief Applications to Pathology, Hygiene, and Forensic Medicine. Especially designed for the Use of Students. 2nd Edition. By *W. B. Carpenter*, M.D. F.R.S.

If it were practicable for us to carry our readers back to the state of physiological knowledge twenty years ago, as it was displayed in the teaching of the schools and in the writings of authors, the satisfactory truth would become apparent, that not only has physiology with its inseparable attendants, comparative and minute anatomy, kept pace with the rapid march of modern science, but, which must be regarded as an infinitely more important matter, that a much more philosophic spirit has been infused into this and all kindred researches. Physiologists have at length perceived that the actions of living bodies are to be studied in accordance with the same mode of procedure, as that adopted in the investigation of all other natural phenomena, of which procedure careful and extended observation must ever be regarded as the essential basis. They have, without entering into a compact similar to that of the geologists, who, vividly impressed with the evils resulting from mere speculation and slavish adherence to system, in founding their admirable society, announced its great object to be to encourage and record observations; the physiologists we say, have tacitly come to a similar agreement, and rich and valuable have already been the results. Ceasing to be what Cuvier terms "Naturalistes de Cabinet" they have become *observers*—instead of continuing to speculate upon the respective shares of nervous power and physical forces in supporting the operations of living bodies, they have contented themselves with ascertaining facts; and more than all, in place of regarding the phenomena of the animal economy as of a bizarre and mysterious character altogether sui generis, they have submitted them to the test of experiment, with the conviction that, although many of the vital actions are undoubtedly peculiar in their nature, yet as all of them occur in material substances, or, to borrow the happy expression of Dr. Arnott, as "life is a superstructure on physics and chemistry," the ordinary laws of matter must exert potential influence. The more the inquiry is extended, the more apparent will become the necessity of keeping prominently in view this great truth, which, in the sphere of the organic functions especially, is the very clue of the whole inquiry.

The general result of this improved mode of cultivating physiological science, has been to introduce more certain knowledge into all its branches; to substitute for vague surmises clear and definite notions; and by the aid of minute, developmental, and comparative anatomy, to establish the uniformity of the laws of organization. When such have been the fruits obtained in less than a quarter of a century, we may affirm, without indulging in vain and sanguine anticipations, that by a perseverance in the same path, the time is not distant when the science of life, surpassing as it does in absorbing interest all other branches of temporal knowledge, will, in the number of its facts and the soundness of its generalizations, closely approach the purely physical sciences—astronomy—mechanics—and chemistry.

Although we may be thus hopeful for the more philosophical branches of medical science, it cannot have escaped the attention of those who have watched the progress to which we have alluded, that hitherto few practical improvements of marked importance have resulted from the laborious researches of the last few years. The absence of such improvements have, we are aware, been urged by many men of eminence as a proof of the inutility of minute observations, and some think that much valuable time and talent are being wasted by the zealous cultivators of organization in the present day. In this opinion we do not participate; firstly, because the history of all the great achievements by which the convenience, the comfort, and the happiness of mankind have been secured, shows that the discoveries and labours of such men as Kepler, Hooke, and Black, barren and unprofitable as they appeared to their contemporaries, were the fruitful and necessary precursors to a multitude of subsequent practical appliances; and, secondly, because we contend that all which increase the scope of the intellect, and stores it with truths of a philosophic character, although possibly having no direct bearing upon the treatment of disease, is of higher import than any equal amount of technical information, inasmuch as it strengthens, invigorates, and disciplines that which is the source of all knowledge, the mind itself. We may perhaps be biassed in this matter, but considering the ample time which pupils are now required to devote to study, we regard it as a circumstance full of promise for future excellence and future progress, that chemistry, inorganic and organic; philosophic anatomy—vegetable, animal, and comparative; and scientific physiology, constituting, as it were, the mathematics of medical science, are now zealously and ably cultivated in our various schools.

It is in such a spirit and with such objects that the admirable work of Dr. Carpenter has been written; and it affords us much pleasure to find that it has so speedily reached a second edition ; not only for the sake of its talented and industrious author, but more especially because, taken in connexion with the successful progress of other similar works, this circumstance may be received as a sure indication of a more elevated taste pervading the professional mind. As the former notice of Dr. Carpenter's work (*see Review, July* 1843) was from unavoidable circumstances necessarily incomplete, it will be our object in the present article to present an epitome of the contents of the volume before us, and also to add, when it may appear necessary, some of the more important facts derivable from other sources.

The arrangement of the functions is the same in the present edition as in the former one ; and although we agree with the author that this is a matter of subordinate importance, yet as it seems to us desirable never unnecessarily to disturb the beautiful subordination and harmony evinced in the works of the Creation, we should have preferred, as being more in keeping with the natural relations of the nutritive and sentient phenomena, and we may add with the scientific character of the work itself, if those processes which are essential to all life, such as absorption—respiration and assimilation, and on which the special actions of animal existence repose, had been in the first place investigated. The formation and actions of the human organism display such exactness, are submitted to such undeviating laws, and observe so definite a sequence, that in departing from the natural order the student soon finds himself in a labyrinth of intricacies and involvement. Dr. Carpenter has however avoided this inconvenience to a great extent, by prefixing to the account of the nervous system, a concise but comprehensive sketch of the functions, vegetative and animal, and of their mutual relations.

The advances of science have enabled the author to make considerable additions to the Second Edition. The following comprise the principal novelties.

"1. The researches of Mr. Newport (confirmatory, in every important particular, of the Author's previously expressed views) on the structure and functions of the double Nervous cord of the Articulata, §§ 143, 146.

"2. Dr. Stirling's observations on the Spinal Cord, § 165.

"3. A more precise form given to the Author's own views, on the localization of the Emotional and Instinctive actions, §§ 259—265.

"4. Observations on the Nature and Destination of the Food of Animals; principally based on Prof. Liebig's views, §§ 430—434.

"5. Mr. Goodsir's researches on the absorbent Cells of the Intestinal Villi, §§ 461, 462.

"6. Researches of Mr. Addison and M. Bourgery on the structure of the Lungs, § 525.

"7. Researches of MM. Andral and Gavarret, and of Prof. Scharling, on Respiration, § 534 *note*, and § 544.

"8. The chapter on Nutrition, including the history of the Chyle, Blood, and Tissues, almost wholly re-written; so as to include the results of the most recent observations on these subjects, and the views to which the Author has been led by them.

"9. The doctrine of Secretion by Cells, as developed by Henle, Goodsir, and others, § 651.

"10. The researches of Mr. Bowman on the structure and functions of the Malpighian bodies of the Kidney, §§ 667, 668.

"11. The account of the constitution of Urine lately given by Liebig, §§ 673—678.

"12. The inquiries of Dr. Lehmann, respecting the influence of diet on the constitution of the Urinary Secretion, §§ 679, 680.

"13. The researches of Dr. Evans on the structure of the Spleen, §§ 708, 709.

"14. The inquiries of Dr. Buchanan on the changes in the Blood produced by admixture of Chyle, §§ 714 *note*, 715.

"15. The investigations of Chossat on the effects of Inanition, § 730.

"16. The results of M. Raciborski's inquiries, in regard to the relation between the periods of Conception and Menstruation, § 742.

"17. The researches of Dr. Ritchie into the various conditions of the Ovaria, § 744."—*Preface*, p. 7.

After a brief exposition of the connexion of physiology with the other branches of medicine, the author, in order to fix the position of man in the zoological scale, and at the same time to trace the successive development and correlation of the various organs and functions as they make their appearance in the animal series, gives a comprehensive and well digested account of the several classes of the avertebrate and vertebrate divisions of the animal kingdom. In contrasting the internal and true skeleton of the vertebrata with the external skeleton as it is usually called of the invertebrate classes, Dr. Carpenter has a passage, which unexplained, is likely to mislead as to the true relations of the parts in question. "The skeletons, he remarks (p. 15), of most of the invertebrata differ however from those of vertebrate animals in this important character, that they are not permeated by vessels, and are formed only by a superficial deposition." Although the author is of course himself aware of the real homology of these organs, yet it may not here be superfluous to point out that the outer covering of the body of radiate, articulate, and molluscous animals, whether consisting of a soft and yielding tegument, as in the common earth-worm, of a horny or calcareous envelope as in insects and crustaceans, or of a testaceous covering, as in the conchifera, is the corresponding part, not of the skeleton, but of the epidermis of the vertebrata. Like the epidermis, these coverings are in many instances repeatedly cast off, as in the metamorphoses of insects, and in the periodical change of the shells of crustacea. In many of the more minute animals, an entire identity may even be detected; in the monoculus, for example, a small fresh water crustacean, the external covering consists of a delicate epithelium, the cells of which, when viewed by the microscope, closely resemble epidermic scales; the same thing may be also observed in the familiar microscopic object called the skeleton larva.

It is in fact well known that, with various modifications, all animals possessing a distinct nervous system, have two skeletons:—the *neuro-skeleton*, often reduced to a fibrous membrane, as generally happens in the avertebrata,* and even in a few instances in the vertebrata, as in the case

* It is stated by Dr. Will (Müller's Archiv. 1844, p. 77,) that, in the avertebrate animals, the central parts of the nervous system, are covered by two membranes, of which the exterior one, in some instances, as in the cray-fish and of the myxinoid fishes and amphioxus, surrounding the nervous centres; and a *dermo-skeleton* covering the surface of the body. One of the most interesting and instructive examples of this combination is afforded by the highest class of mollusks, the cephalopoda, in which, as in the nautilus pompilius, a rudimentary cranium, &c. in the state of cartilage, and an external shell, are met with in the same individual.

In considering the essential character of the skeleton of the vertebrata, the author well observes, we should look at its simplest forms—those in which it has the least number of superadded parts. This is a maxim of universal application in the study of organized bodies, for as the laws of Nature are fixed and definite, we may always expect to find that which constitutes the real and typical character of an organ, revealed in the class of animals in which it first makes its appearance.

Applying this test in the case before us, we find the skeleton in the serpent tribe among reptiles, and in the eel and its allies among fishes, consisting of the spinal column with the cranium at its anterior extremity, and this evidently developed in reference to that which it is mainly formed to protect, the nervous centres, consisting of the brain and the spinal cord. The bone called a vertebra is then the typical part of the skeleton, for, as our author observes, " the cranium is in reality formed of the same elements as the vertebræ" : all other parts—limbs, chest, and pelvis, being subordinate, may or may not exist.

An interesting comparison is made by Dr. Carpenter, between the typical character of the articulated and molluscous animals and those of the vertebrata, which we regret our limits will not allow us to insert.

The existence of rudimentary lungs in the swimming bladder of the fish (a fact inferred by Hunter)—the combination in the batrachia of lungs and gills—and the now well-known fact that many other animals besides tadpoles and insects undergo a metamorphosis, are pointed out.

We must confine our notice of the general character of mammalia and man, to the following sketch of the leading peculiarities by which the human organism is distinguished from that of animals.

" Man cannot be regarded as distinguished from other Mammalia, however, either by acuteness of sensibility, or by muscular power. His swiftness in running, and agility in leaping, are inferior to that of other animals of his size,—the full-grown Orang for example. The smallness of his face, compared with that of the cranium, shows that the portion of the nervous system distributed to the organs of sense, is less developed in him than it is in most other animals; and the small proportional size of the ganglionic centres, with which these organs are immediately connected, is another indication of the same fact. Accordingly, he is surpassed by many in the acuteness of his sensibility to light, sound, &c.; but he stands alone in the power of *comparing* his sensations, and of drawing conclusions from them. Moreover, although none of his senses are very acute in his natural state, they are all moderately so, which is not the case in other animals; and they are capable (as is also his swiftness of foot) of being much improved by practice, especially when circumstances strongly call for their exercise. This power of adaptation to varieties in external conditions, which makes him

---

gasteropods, consists of a peculiar cellular tissue, whilst, in other cases, as in the leeches and insects, it is composed of the ordinary cellular tissue condensed around the ganglia.

to a great extent independent of them, is manifested in other features of his structure and economy. He is capable of sustaining the lowest, as well as the highest, extremes of temperature and of atmospheric pressure. In the former of these particulars, he is strikingly contrasted with the anthropoid Apes, such as the Chimpanzee, which is restricted to a few of the hottest parts of Africa, and the Orang Outan, which is only found in Borneo and Sumatra: these cannot be kept alive in temperate climates, without the assistance of artificial heat; and even when this is afforded, they speedily become diseased and die. His diet is naturally of a mixed kind; but he can support himself in health and strength, on either animal or vegetable food exclusively. It is by the demands which his peculiar condition makes upon the exercise of his ingenuity, that his mental powers are first called into active operation; but, when once aroused, their development has no assignable limit. The slow growth of Man, and the length of time during which he remains in a state of dependence upon his parents, have been already mentioned as peculiarities, by which he is distinguished from all other animals. He is unable to seek is own food, during at least the three first years of his life; and he does not attain to his full stature, until he is more than twenty years of age. In proportion to his size, too, the whole sum of his life is greater than that of other Mammalia. The greatest age of the Horse, for example, which is an animal of much superior bulk, is between thirty and forty years. That of the Orang, which, when full grown, surpasses Man in stature, is about the same, so far as can be ascertained. The age to which the life of Man is frequently prolonged, is well known to be above a hundred years; and instances of such longevity are to be found in all nations." P. 54.

In the Second Chapter a succinct account is given of the functions or vital actions, and of their mutual relations, and to this part of the work we would call particular attention. "The idea of life or vital action obviously involves that of change. We do not consider any being as alive which is not undergoing some continual alteration perceptible to the senses."

Thus—"If we contemplate the history of the Life of a Plant, we perceive that it grows from a germ to a fabric of sometimes gigantic size,—generates a large quantity of organised structure, and many organic compounds, which form the products of secretion but do not undergo organisation,—multiplies its species, by the production of germs similar to that from which it originated; but that it performs all these complex operations, without (so far as we can perceive) either feeling or thinking, without consciousness or will. All the functions of which its Life is composed, are, therefore, grouped together under the general designation of Functions of *Organic* or *Vegetative* life; and they are subdivided into those concerned in the maintenance of the structure of the individual, which are termed functions of *Nutrition*,—and those to which the *Reproduction* of the species is due.

"On analysing the operations which take place in the Animal body, we find that a large number of them are essentially the same in character with the foregoing, and differ only in the conditions under which they are performed; and that we may, in fact, readily separate the Organic functions, which are directly concerned in the maintenance of the fabric, from those of Animal life, the chief purpose of which is entirely different." P. 59.

In this latter passage is expressed the true nature of the nutritive processes of animals, which are essentially the same as those of plants, but on the whole infinitely more active, in consequence of the great law impressed upon organic beings, that all vital action causes a waste or disintegration of the instruments or organs employed. The incessant molecular change of the animal frame has been known from the earliest periods, and there can be no doubt, as Dr. Carpenter infers, that this destructive process is not confined to the muscles, of which only Liebig in his celebrated treatise on Organic Chemistry has treated, but also occurs in the nervous and all other tissues, even the most dense, as the granules of bone and the ivory of the teeth. That admirable reasoner, Bishop Butler, has founded upon this incessant change of the nervous tissue a beautiful argument, that as the spiritual part of our nature is not affected by the gradual disintegration which is incessantly proceeding in the brain, and by which it is clear that in a given time the whole of it is changed, so, in like manner, it is not difficult to conceive of the soul surviving the sudden and total destruction of that organ, the question resolving itself simply into one of degree, not of kind.

As we shall, in a succeeding number of our Journal, consider the additions which have lately been made to the physiology of the nervous system, we proceed to Chap. IV. which has for its title, "On Sensation and the Organs of the Senses."

" By the term *Sensation* is rightly understood that change in the condition of the mind, by which we become aware of an *impression* made upon some part of the body; or, in a briefer form of expression, it may be defined to be the *consciousness of an impression.* Some physiologists have, it is true, spoken of a *sensation without consciousness;* but it seems very desirable thus to limit the term; since the word *impression* may be very well applied to designate the change produced in the afferent nerves by an external cause, up to the point at which the mind becomes conscious of it. We have seen reason to believe, that the impressions communicated to the Spinal Cord may there excite motor actions, without occasioning true Sensation; and it would seem to be with the Brain only that the mind possesses the relation necessary for the production of such a change in it. Hence the Brain is spoken of as the *Sensorium.*" P. 249.

We entirely coincide in the correctness of this definition, and in the necessity of limiting sensation to the brain. It has always appeared to us a profound error in physiology to admit any kind of sensation of which the mind is not conscious: indeed, the very notion of the phenomenon implies an act of consciousness, and when the presence of the latter cannot be proved or reasonably be inferred, it is only unnecessarily embarrassing the question to contend for the former.

In speaking of the connexion between the capillary circulation and nervous action, the author has, in our opinion, over-estimated the quantity of blood furnished to the nerves, an error very likely to arise in consequence of anatomists having drawn their conclusions from observations made principally on the tactile papillæ of the fingers. These parts are, it is true, provided with very rich vascular plexuses, but it must be remembered that a large proportion of the blood thus distributed is not for the supply of the nerves but of the secretory organs of the skin; a fact which is strikingly shown by the existence of a vast multitude of linear vascular tufts beneath the nail, where the nervous twigs are comparatively few in number and the sensation dull. If a trunk of a nerve be injected and be then examined microscopically, the vascularity is seen to be but moderate. The only parts of the nervous system which are in reality very richly supplied with blood, are the active centres—the brain—the spinal cord, and the sympathetic ganglia.

The following observations are so expressive of what must ever be regarded as a fundamental law of sensation that we give the passage entire.

"It may hence be inferred that no nerve of *special* sensation can, by any possibility, take on the function of another. How far the nerves of *common* sensation can, under any circumstances, perform the offices usually delegated to those of special sense, we are not yet in a condition to determine. Comparative Anatomy seems to show that, in the lowest animals in which the rudiments of eyes can be detected, there is no distinction between the nerves proceeding to these organs, and the rest; and there would appear some ground for the belief that, as in other cases, the special organs of sensibility are gradually elaborated, in ascending the Animal scale, from the more general apparatus, and are not merely superadded to it. Hence we may conceive the possibility (though there is no proof of the fact) that states of the system might occur, in which a change in the common sensory nerves might produce the sensation of light, sound, &c. But it is quite impossible (so far at least as our present knowledge of physical phenomena permits us to decide upon the impossibility of anything) that distinct visual impressions should be communicated to a nerve, except through the mediation of such an optical instrument as the eye; or distinct sonorous impressions, except through such an acoustic instrument as the ear. Hence we must receive with the greatest caution the wonderful accounts of transference of sensation, many of which have undoubtedly been the offspring of deception. Still it may be objected that, as we are so totally destitute of real knowledge, as to the mode in which vision is ordinarily produced by inverted images upon the retina, we have no right to assert that it *may not* take place in some other way; and perhaps this objection should lead us to consider the phenomenon rather as *extremely improbable*, than as *impossible*. But the improbability may be compared to that of a stone ascending like a balloon, or a piece of lead floating on the water; for we have no more knowledge of the ultimate cause of that which we term the force of Gravitation, than we have of the nature of Sensation." P. 257.

So strictly is this the case that each nerve of sense, whenever and however excited, never produces any other than its own special action; thus, if the retina be struck, a flash of light is perceived—if the auditory nerve be stimulated by a congested or inflammatory state of its blood-vessels, a singing or rumbling noise is the result—if the nerves of the skin be excited, in whatever manner, a sensation of touch is caused. These, and a multitude of other similar phenomena more particularly connected with what are termed *subjective sensations*, demonstrate, at least if any process of inductive argument is to be deemed conclusive, that clairvoyance in all its shades and shapes is a physiological impossibility.

In the section upon touch, a reference is made to the interesting facts ascertained by that admirable and sound anatomist Professor Weber,* and which are well worthy of careful perusal. (P. 262.)

In connexion with this subject, Dr. Carpenter well remarks "that the conditions under which certain of the modifications of common sensation

* We have often regretted that the comprehensive and standard work of Hildebrandt, Handbuch der Anatomie des Menschen, edited and greatly improved by Prof. G. H. Weber, has not been rendered available, by translation, to the English student.

operate, are in some respects different from those of ordinary Touch is very easily shown. Thus, the feeling of tickling is excited most readily in parts which have the least tactual sensibility, the armpits, flanks, and soles of the feet, whilst in the points of the fingers it cannot be excited." The fact also that the susceptibility of the surface to temperature does not correspond to the acuteness of touch is pointed out, and in connexion with this point we would remark, that certain anomalous instances of local loss of sensation, would almost induce us to infer that the power, if not the nervous fibrils themselves, by which we judge of variations of temperature, is altogether distinct from the perception of mere contact. A case confirmatory of this view was related to us by an intelligent practitioner, of a patient who, although he had lost the sensation of touch in the skin of one of the lower extremities, so that he was not conscious of contact, still retained in the affected part the perception of heat and cold.

The sense of taste, according to the author, ought to be considered as a peculiar modification of that of touch, but in this position we do not agree. The common experience of mankind might, we think, be adduced to show that these two faculties are essentially distinct in kind. The difference in the ultimate texture of nerves having undoubtedly separate offices, is not so marked as to enable us to pronounce upon the functions of a part simply upon the grounds of anatomy; and, consequently, the fact of a part of the fifth pair supplying the organ of taste, for we regard, notwithstanding the experiments of Panizza, the lingual to be the true nerve of taste, is not sufficient to disprove the existence of special fibres being allotted to that sense.

The sections on the senses of Vision and Hearing are written in a philosophic spirit, and are worthy of careful consideration, but some space having been devoted to these subjects in a former review, we can only here quote the following passage illustrative of the complex character of what, in the absence of such evidence, might be regarded as simple ideas.

> "The sense of Vision depends, in the first place, on the transference to our minds of the picture which is formed upon the retina; this picture puts us in possession of the outlines, lights and shades, colours and relative positions, of the objects before us: and all the ideas respecting the real forms, distances, &c. of bodies, which we found upon these data, must be considered in the light of *perceptions* either instinctive or acquired. Many of these are derived through the combination, in our minds, of the visual sensations, with those derived from the sense of touch. Thus, to take a most simple illustration, the idea of *smoothness* is one essentially tactile; and yet it constantly occurs to us, on looking at a surface which reflects light in a particular manner. But, if it were not for the association, which experience leads us to form, of the connection between *polish* as seen by the *eye*, and *smoothness* as felt by the *touch*, we should not be able to determine, as we now can do, the existence of both these qualities, from an impression communicated to us through either sense singly." P. 277.

In treating of the muscular system, the author has availed himself of the excellent researches of Mr. Bowman; but we regret that, in such a treatise, no reference is made to the important researches of Matteucci, upon muscular contractility.

The second division of the work comprehends the organic functions to which, in the present edition, considerable additions have been made.

In the history of the digestive process the celebrated and well-known investigations of Dr. Beaumont are throughout adopted. The observations of Mr. Goodsir on the structure and action of the intestinal villi, are thus recorded and adopted.

"From the recent observations of Mr. Goodsir, it appears that the villi are enclosed in a very delicate membrane (analogous to that which lies under the epidermis and epithelium in the skin and mucous membrane); and that, when digestion is not going on, they are covered by an epithelium. The space between the reticulations of the blood-vessels and lymphatics, towards the extremity of each villus, is occupied, whilst the absorption of chyle is taking place, by a number of spherical vesicles or cells, varying in diameter from the 1-1000th to 1-2000th of an inch, and containing an opalescent fluid. At the part where the vesicles approach the granular texture of the substance of the villus, minute granular or oily particles are seen. When the intestine contains no more chyme, the vesicles disappear almost entirely, the lacteals empty themselves, and the villi become flaccid; the epithelium, which had fallen off during the process of Absorption, is then renewed. The vesicles at the extremities of the villi can scarcely be regarded in any other light than as *cells,* whose lives have but a very brief duration,—selecting from the materials in contact with the surface of the villi, and appropriating these to their own growth,—then liberating them, by solution or disruption of the cell-wall, in a situation where they can be absorbed by the lacteals." P. 395.

In connexion with cutaneous absorption, some interesting examples are quoted from different writers, showing the great activity and importance of the process. Dr. S. Smith mentions that a man who had lost nearly three pounds by perspiration during an hour and a quarter's labour in a very hot atmosphere, regained eight ounces by immersion in a warm bath for half an hour. Dr. Dill relates the case of a diabetic patient who, for five weeks, passed 24 lbs. of urine every twenty-four hours, his ingesta during the same period amounting to 22 lbs.; and as he had lost in weight only 27 lbs. when he died, there must have been an absorption of 43 lbs. from the atmosphere during the time.

In treating of the respiratory organs the author truly observes that "in regard to the intimate structure of the lungs of Man and of the Mammalia it is difficult to speak with confidence."

The account of Reisseissen, according to which the air-cells are merely the globular dilatations of the ultimate ramifications of the bronchial tubes, is certainly erroneous: nor is the opinion of Mr. Addison much more satisfactory, that the air-cells do not exist prior to birth, but that they are produced by the parietes of the minute bronchial tubes becoming dilated into little pouches, in consequence of the pressure exerted by the atmospheric air admitted into the lungs in respiration.

An excellent paper upon this subject, containing the results of a careful examination of the pulmonary texture, made by Mr. Rainey, of St. Thomas's Hospital, has been lately read before the Medico-Chirurgical Society; and as these investigations, in addition to affording clear and satisfactory evidence upon many points which are at present a matter of dispute and uncertainty, contain some novel facts, a brief allusion to them will not be misplaced.

The principal results are as follows:—

1. A bronchus, when traced from its commencement to its termination, is seen to be in the first part of its course more or less cartilaginous; it then becomes destitute of cartilage, retaining however a perfectly circular form, and having no air-cells opening into it; farther on, being still circular, numerous air-cells open into it; lastly, the air-cells increase so much in number, and open into the bronchus so closely to one another, that the tube can no longer retain its circular form, but becomes reduced to an irregular passage, running between the cells, and ultimately reaching the surface of the lobule, ends by forming a terminal air-cell.

2. The air-cells are small irregularly-shaped cavities, having generally four or five unequal sides; those which are situated close to the small bronchial passages open into them by well-defined circular apertures, whilst those which are situated at a distance from these passages open one into the other, an arrangement which those who are acquainted with the disposition of the air-cells in the injected lung of the frog and serpent will readily comprehend; in fact, *each lobule of the lung of the mammal and man, with its bronchial passages and appended cells, may in some sort be regarded as a repetition of the whole lung of the frog.*

3. The border of each air-cell is surrounded, in addition to the epithelium, by a number of fibres definitely arranged in a circular manner, so as to form a circumscribed limit to each cell. The fibres appear to be elastic, and have no resemblance whatever to muscular fibres, striped or unstriped.

4. The sides or walls of the air-cells consist of a dense plexus of capillaries, situated, in the interior of the lobules, between two layers of the pulmonary membrane; but on their exterior between this membrane and the pleura in the case of the lobules on the outer surface of the lung, or between it and the interlobular areolar tissue in those lobules which bound the interlobular spaces. There is thus, between every two cells only one vascular network, so that the small stream of blood in each capillary vessel is acted on by the air upon both sides, whilst in the frog, serpent, &c. there being two plexuses of vessels between two cells, the blood in the capillaries is only aerated on one side.

5. The number of capillary plexuses is not the same as that of the air-cells, one net-work passing between and supplying several cells; or, in other words, one terminal branch of the pulmonary artery supplies the plexuses of several air-cells.

6. An important part of these investigations relates to the anatomy of the fœtal lungs, prior to the act of respiration; these, when well injected, are distinctly seen to possess air-cells, fully formed and surrounded, as in the animal which has respired, by plexuses of blood-vessels.

As we trust that the whole of this valuable paper will be speedily published, we do not notice the interesting observations of Mr. Rainey upon the seat and effects of tubercle.

Dr. Carpenter alludes to the researches of M. Bourgery upon the force of the respiratory muscles, and the capacity of the lungs at different periods of life and in the two sexes. According to these inquiries the development of the air-cells continues up to the age of 30, at which time the capacity of respiration is the greatest; it subsequently decreases, especially in persons who suffer from cough, the violence of such expiratory effort frequently causes rupture of the air-cells, and thus gradually produces that emphysematous state of the lungs, which is so common in elderly persons. The power of increasing the volume of air by a forced inspiration is much greater in young than in old persons, and is twice as great in males than in females of the same age, a circumstance which is evidently connected with the extent to which muscular efforts can be carried in these classes respectively.

Since the work before us was written, a valuable contribution to the physiology of the respiratory muscles and the capacity of the lungs, has been made by Mr. Hutchinson, in a paper read before the Statistical Society, and subsequently published in their Journal, for September, 1844. The deductions of this gentleman rest upon an extended basis, and the results being tabulated and accompanied with illustrative diagrams, are readily comprehended.

By the term "*capacity*" Mr. Hutchinson "signifies that quantity of air which an individual can force out of his chest by the greatest voluntary expiration, after the greatest voluntary inspiration." The experiments respecting "capacity," were made with the assistance of an instrument called the *spirometer*, or "breath-meter," and those on the force of the muscles by a mercurial guage.

It has long been known that the quantity of air capable of being admitted into the lungs varies in different individuals, and these researches have established this in a remarkable degree, showing the modifying effects of stature and occupation, and of health and disease. Thus, the mean "capacity" of 172 males under the height of 5 feet 8 inches is 220 cubic inches, whilst that of 82 males of from 5 feet 11 inches to 6 feet, is 255 cubic inches. It is clearly a desirable thing to discover the cause of such a marked difference, and Mr. Hutchinson asserts that he has "discovered a relation intimately existing between this capacity and power and the *height* of the individual," and further on, states it to be a rule that "*for every inch of height* (from 5 to 6 feet) *eight additional cubic inches of air at* 60° *are given out by a forced expiration.*" The exceptions to this rule among healthy persons, occur among very stout and corpulent individuals, whose capacity stands the lowest. This, if confirmed, is a very interesting generalization, and strongly supports, both by the rule and its exception, the chemical theory of animal temperature; for it thus appears that the activity of the heating apparatus, the lungs, is definitively proportioned to the bulk of the body to be warmed, except in the case of fat persons, in whom a thick layer of adipose matter, which is a bad conductor of heat, exists on the surface of the body.

Another curious and unexpected result is, that the force of the expiratory muscles is about one-third stronger than that of the inspiratory; thus whilst a person of 5 feet 6 inches elevates the mercury 3·87 inches by the power of his expiration, he only raises it 2·70 inches by his inspiration. This difference depends probably more upon the favourable leverage of the abdominal muscles on the fore part of the trunk, and the erectores spinæ behind, than upon the actual size of those muscles when contrasted with the diaphragm, pectorales, serrati, and the other muscles of the inspiratory class.

The effects of employment, diet, and disease are strikingly shown; for example, in the early stage of phthisis the healthy "capacity" being

204 cubic inches, the capacity of the diseased lungs was in some cases 135 cubic inches; and in the advanced stage, as would be anticipated from the evidence of morbid anatomy, the difference becomes much greater, so that the "capacity" of a person which in health would be 229 cubic inches, was reduced to 80 cubic inches. Mr. Hutchinson conceives that his tests afford an additional and important means of detecting the existence and extent of pulmonary disease; and even of discovering other affections, such as hernia and perforation of the membrana tympani, seated in organs indirectly related to the respiratory apparatus.

The chapter on Nutrition having been, as the author states, almost entirely re-written, contains the most recent inquiries upon the subject. The admirable researches of Dr. Prout, since confirmed and extended by the German school, have shown that all the azotised parts of the animal body are derived from one peculiar proximate principle, called by Prout *albumen*, and by Mulder *protein;* if to this we add fatty matter, certain salts, and water, we have all the materials required for the nutrition of animals. In illustration of his views, Dr. Prout has selected milk as an example, observing that "milk is designed and prepared by nature expressly as food, and it is the *only material* throughout the range of organization that is so prepared. In milk, therefore, we should expect to find a model of what an alimentary substance ought to be—a kind of prototype, as it were, of nutritious matter in general."

We have ourselves, however, always considered the ovum as a case more in point, on account of its containing only one protein compound, albumen, and this is the instance adduced by Dr. Carpenter. From the albuminous matter of the egg of the fowl all the various and complex organs are derived, the first and most essential part of the process consisting in the conversion of that substance into fibrin, which is to be regarded as the *plastic* or *organizable* constituent of the body. "To use a rather homely illustration, albumen, fibrin and organized tissue, stand much in the same relation to each other, with raw cotton, spun yarn, and the woven fabric." Now the conversion here alluded to, is, we must presume, effected by the agency of those important organic instruments called *nucleated cells*, of the powers of which the author has given a full account in the several divisions of his work relating to the nutritive actions.

There is no discovery of modern times which has worked such mighty changes in established opinions, as that connected with the *cell theory*, which, originating in the researches of Schleiden into the ultimate texture of vegetables, was extended in the celebrated work of Schwann (Mikroskopische Untersuchungen, &c.) to the organization of animal structures. By these investigations, it has been ascertained, that with some few exceptional cases, every part of every animal and plant is, in the first epoch of its formation, developed from a nucleated cell; that by the inherent and independent powers of this cell and by the metamorphoses it experiences all the organic tissues are formed; and, more than all, that every act of the process of nutrition—secretion—absorption—assimilation—growth, and decay, instead of being, as until this theory they were in all the higher animals invariably considered to be, immediately dependent upon the blood-vessels and absorbents, are in reality accomplished by parts which are essentially extra-vascular. Although the facts here announced have received the concurrent sanction of all accurate observers in every part of Europe, yet as we have reason to know that among many of our professional brethren, a lingering attachment to former doctrines, and an indisposition to admit the somewhat startling deductions which have flowed from the observations of Schwann and his numerous followers, still prevail, a few remarks on the subject may not be misplaced.

When we recal to mind the extreme simplicity, the undeviating uniformity, the mathematical exactness of all the great laws of Nature, it is not difficult to comprehend that the mechanism by which one great class of vital actions, the nutritive, are sustained, should present a common type. The humblest of the cellular plants displays phenomena which are essentially the same as those which in the aggregate constitute the organic life of the most complex animal:—it absorbs nutriment—it grows—it reproduces, and thus maintains its own existence and that of its species. A similar series of actions are performed in the simplest classes of the animal creation, and in both instances the only organs which can be detected are—cells. Now, if we turn to the most elaborately constructed animal, to the human body itself, what do we find? The embryo, as we learn from Martin Barry's admirable investigations, taking its rise from a single cell—the formation of a membrane, the vesicula germinativa—the development to a considerable extent of the nervous centres—the deposition of the commencing vertebral column—all these phenomena occurring antecedently to the appearance of blood-vessels and of their central organ, the so-called punctum saliens. If from the nutritive process of the embryo we turn to that of the adult, the same kind of evidence is afforded of the predominant action of cells, and the subordinate agency of vessels. Thus the active and essential part of every organ, that in fact which constitutes the organ, is extra-vascular. No artery penetrates the sarcolemma, the ultimate nervous tubule, or the efficient agent of the various glands. Again, few organs appear to be more vascular than the mucous coat of the small intestine; and yet here the parts engaged in the secretion of mucus and the absorption of the chyle, are seen to consist of epithelial cells: even the more simple membranes, the serous and the synovial, those in which, if any where, the direct action of the capillary vessels might be expected, present in the place of the exhalent arteries of former anatomists, a layer of delicate epithelial cells, the true agents of the secretion.

All this appears somewhat startling, but when the matter is presented to our notice in its simplest form, the truth of it becomes obvious:—for example, the animal machine consists in addition to blood-vessels of several different solid substances, muscle—nerve—bone. Now as two bodies cannot occupy the same space at the same time, it plainly follows that all these substances must be placed beyond the tubes carrying the blood, and this fact being once understood, all the details respecting the degrees and relations of vascularity become questions of secondary importance. The axiom, therefore, that organization is co-extensive with, and dependent upon vascularity, must henceforth be abandoned, and with it will vanish a crowd of errors and apparent anomalies respecting the vitality of parts wanting blood, such as the epidermis and cartilage in vertebrated animals, and the entire organism of many of the more simple classes among the invertebrata. We will only further remark, that the great principle thus established, that throughout the organic creation, the nutritive process is effected by one typical formation, the nucleated cell, is a generalization which promises to effect for structural anatomy, what the law of correlation has accomplished for comparative anatomy, and that of definite proportions for the science of chemistry.

Dr. Carpenter, who has adopted in all their extent the views at which we have glanced, regarding cells or vesicles as the primordia of all vegetable and animal tissues, gives a comprehensive account of the subject, from which, however, we can only extract one or two passages.

"A very large proportion of the Vegetable Organism (in the simplest Plants, the entire structure) is made up of *cells* or *vesicles;* which are minute closed sacs, whose walls are composed in the first instance of a delicate membrane, frequently strengthened, at a period long subsequent to their first formation, by some internal deposit. The form of these cells is extremely variable; and depends chiefly upon the degree and direction of the pressure, to which they may have been subjected at the period of their origin, and subsequently to it. Sometimes they are spheroidal; sometimes cubical or prismatic; sometimes cylindrical; and sometimes very much prolonged. These cells may undergo various transformations.—One of the most common, is the conversion of several into a continuous tube or Duct.

"The Animal body exhibits phenomena, of a character essentially the same. Even in the fully-formed organism, many parts may be found, which are composed, more or less evidently, of isolated cells or vesicles, analogous to those of Plants; and it has been clearly proved that, in its early condition, the whole fabric has this character. In fact, it has been shown by the researches of Barry, Schwann, and Valentin, that the whole structure originates in a single cell; that this cell gives birth to others analogous to itself, and these again to many future generations; and that all the varied tissues of the Animal body are developed from these, although no difference can be in the first instance observed among them.

"From what has been stated, it appears evident that the process of Nutrition mainly consists in the growth of the individual cells composing the fabric; and that these derive their support from the organic compounds with which they are supplied by the blood, just as the cells composing the simplest Plants derive theirs from the inorganic elements which surround them: and as different species of the latter select and combine these, in such modes and proportions, as to give rise to organisms of very diversified forms and properties, so is it easily intelligible that the different parts of the fabric of the highest Animals should exercise a similar selective power, in regard to the materials with which the blood supplies them. The structure composing every separate portion of the body has (what may be termed) a *special affinity* for some particular constituents of the blood; causing it to abstract from that fluid, and to convert into its own substance, certain of its elements. The conversion is termed *Assimilation.*" P. 487.

The principal novelty contained in the account of the blood, relates to the *white*, or more correctly speaking the *colourless* corpuscles, which it is inferred by the author, exercise a power of assimilation or conversion, by transforming the aplastic into the plastic material, or, in other words, albumen into fibrin. It may be well to premise that in addition to the well-known red particles, the circulating fluid of man and all vertebrate animals contain colourless corpuscles called lymph globules, and, according to Donné and Gulliver, certain minute spheres, called by the former *globulins.* In the avertebrate animals, although there are no red discs, the blood contains a number of colourless corpuscles, which were thought by Hewson, who clearly describes them, to be "similar in shape to those of the blood of more perfect animals, but differing in colour;" whilst by Wagner, whose views are adopted by the author, they are regarded as being analogous with the colourless or lymph corpuscles of the vertebrata.

Dr. Carpenter's reasons for concluding that the conversion of albumen into fibrin is effected by the colourless rather than by the red corpuscles are principally these :—

1. That the process of nutrition is essentially the same in the avertebrate as in the vertebrate animals, and yet in the former there are only white or colourless corpuscles.

2. That the chyle in which the formation of fibrin takes place, contains white corpuscles.

3. That in very young embryos, where the process of assimilation must be most active, the white corpuscles are, according to the observation of Mr. Gulliver, nearly as numerous as the red particles.

4. That in inflammation, where the quantity of fibrin is well known to be remarkably increased, a great accumulation of white corpuscles takes place in the vessels of the inflamed part, as Mr. Addison, Dr. Williams, Mr. Gulliver and others have proved by microscopic observation.

5. That the number of red corpuscles neither undergoes any decided change in inflammation, nor does it bear a distinct proportion to the activity of the formative process in the different classes of animals.

Lastly, that whilst the red particles are greatly diminished in number by repeated losses of blood, the colourless corpuscles and the fibrin are increased. (Pp. 506, 509.)

The elaborating power thus assigned to the colourless or lymph globules, has been attributed with equal confidence by Mr. W. Jones in this country, and less distinctly by Wagner and Henle in Germany, to the red corpuscles.

As it is not our intention to enter into the merits of these conflicting theories, we shall content ourselves with remarking that there is sufficient evidence to show that both the red and colourless corpuscles ought to be considered as *floating cells:* this we regard as proved by the following facts :—

1. The former in fishes, reptiles, and birds, consist each of a vesicle and central nucleus ; and although in mammals the latter appears to be absent, this circumstance alone is not sufficient to disprove the cell character, inasmuch as the nucleus very commonly disappears in vegetable cells, and because cells may even be primitively formed, though very rarely, without nuclei. 2. The red particles distinctly evince the powers of endosmose and exosmose, as Hewson, without however discovering the real nature of the process, long since proved by repeated experiments. 3. In their first formation they possess in all classes, whatever may be their ultimate form, whether that of a bi-convex ellipse or a bi-concave circular disc, the typical figure of all cells, namely, that of a globe or sphere. 5. The colourless corpuscle invariably consists of a vesicle with a nucleus. We have thus in the blood instruments competent to effect changes in their contents ; and the balance of evidence tends to prove that such changes do really take place in the qualities of the blood and of the chyle whilst circulating in their containing vessels. More than this at present we are not inclined to affirm, but the subject is too important, both as regards the healthy and diseased conditions of the body, to remain long undecided.

The section on the Pathological Changes of the Blood (p. 518) merits a careful perusal; for it is quite clear that, whilst we eschew the fanciful and erroneous parts of the humoral pathology, a more important influence in the production of disease than is usually admitted must be assigned to the blood. In the truth of the following remarks we entirely agree.

"From the part which the Blood performs in the ordinary processes of Nutrition, it cannot be doubted that it undergoes important alterations, when these processes take place in an abnormal manner. These alterations must be sometimes the causes, and sometimes the effects, of the morbid phenomena, which constitute what we term the Disease. Thus, when some local cause, affecting the solid tissues of a certain part of the body, produces Inflammation in them, their normal relation to the blood is altered; the consequence is, that the blood, in passing through them, undergoes a different set of changes from those, for which it is originally adapted; and thus its own character undergoes a change, which soon becomes evident throughout the whole mass of the circulating fluid, and is, in its turn, the cause of morbid phenomena in remote parts of the system. On the other hand, the strong analogy between many Constitutional diseases, and the effects of poisonous agents introduced into the blood, appears clearly to point to the inference, that these diseases are due to the action of some morbific matter, which has been directly introduced into the current of the circulating fluid, and which has affected both its physical and its vital properties.*" P. 518.

The account of the formation of the organic tissues, comprises the latest researches, and presents a somewhat extended summary of this important branch of physiological anatomy. The following is an interesting application of microscopic investigation in a subject which has always puzzled the pathologist.

"It is quite evident that the active *Growth* of the Hair can only take place at its base, where alone it is in connection with the vascular system; but the knowledge of its organised structure enables us to explain many phenomena which were previously obscure. Thus, in the disease termed Plica Polonica, a change takes place in the Hair, which often occurs at a distance from its roots; this change consists in the splitting of the hair into fibres, and the exudation from it of a glutinous substance; and these two causes unite in occasioning that peculiar *matting* of the hair, which has given origin to the name of the disease. It is said that bleeding takes place, in this disease, from the stumps of hairs which are cut off close to the skin; and this may be easily credited, since the increased activity in the formative power of the cells of the hair, must require an increase in their supply of blood. It is very easy to understand, from the analogy of the

* "This doctrine has been recently brought prominently forwards, in a Paper on Symmetrical Diseases, read by Dr. William Budd before the Medico-Chirurgical Society, Dec. 16, 1841. The author ingeniously proves, that the symmetry of many diseases (such as certain forms of cutaneous eruptions, rheumatism, &c.) which do not immediately depend upon external causes, necessarily involves the idea of the conveyance of the morbific agent in the circulating fluid; the palsy produced by lead is a very interesting example, in which the agent is known to be mingled with the blood, and to be deposited in the parts affected, which are generally, if not always, symmetrical."

Cellular Plants in which no vessels exist, how the fluid that is supplied to the base of the hair may find its way upwards; and there seems reason to believe, from the well-known fact of sudden change of colour in the Hair under the influence of strong mental emotions, that, even in its healthy state, fluid secreted at the base may be conveyed to its point." P. 551.

Mr. Toynbee's excellent memoir on Non-vascular Tissues is noticed. This is a question of great interest to practitioners, as it is involved in several classes of disease, especially those of the articular catilages, cornea, and crystalline lens, all of which are, when fully formed and in health, *non-vascular*. The disposition of the blood-vessels in relation with these parts will be readily comprehended by a few extracts.

" The *cellular* (that is the articular) Cartilages are never *penetrated* by vessels in the healthy state, although in certain diseased conditions they become distinctly vascular. They are, however, *surrounded* by Blood-vessels; which form large ampullæ or varicose dilatations at their edges or on their surfaces : and from these the cartilages derive their nourishment by imbibition, in exactly the same manner as the frond of a sea-weed (the structure of which is alike cellular) draws into itself the requisite fluid from the surrounding medium." P. 553.

" At an early period of fœtal life, there is no distinction between the cartilage that is ultimately to become the Osseous Epiphysis, and that which is to remain as Articular Cartilage; both are alike cellular; and the vessels that supply them with nutrient materials penetrate no further than their surfaces. At a subsequent period, however, when the ossification of the epiphysal cartilage is about to commence, vessels are prolonged into it; and a distinct line of demarcation is seen betwixt the *vascular* portion, which is to be converted into Bone, and the *non-vascular* part, which is to remain as Cartilage." P. 554.

" No vessels can be traced (according to Mr. Toynbee) into the substance of the true Cornea; which, contrary to the statement of Müller, is a cellular rather than a fibrous cartilage. The cells are not so numerous as are those of the articular cartilages; and they are surrounded by a plexus of bright fibres, laxly connected together, so as to resemble areolar tissue. Two sets of vessels, a superficial and a deep-seated, surround the margin of the cornea. The arteries of the former are prolonged for a short distance upon the Conjunctival membrane, which forms the outer lamina of the cornea; but they terminate in veins at from $\frac{1}{8}$ to $\frac{1}{2}$ a line from its margin. The deep-seated vessels belong to the cornea proper; but they do not enter it, the arteries terminating in veins just where the tissue of the Sclerotic becomes continuous with that of the Cornea. In diseased conditions of the cornea (as of the articular cartilages), both sets of vessels extend themselves through it; the superficial not unfrequently form a dark band of considerable breadth round its margin; whilst the deep-seated are prolonged into its entire substance." P. 555.

One of the highest authorities in this branch of surgery remarks, that although vessels in ulceration of the cornea frequently become so much enlarged as to admit red blood, " yet there can be no doubt that ulcers do heal without a single red vessel making its appearance." In this latter instance the common opinion is that, as union cannot take place without the co-operation of blood-vessels, minute capillaries, the so-called *vasa serosa*, deposit the plastic substance; but in the present state of our knowledge respecting nutrition, and especially with the facts before us which Mr. Toynbee has established, we are inclined to believe that the reparative process may and does occasionally take place in the cornea without the agency of vessels.

After what we have stated respecting the agency of cells, the facts just quoted, which a few years ago would have seemed to be perfectly anomalous, are readily interpreted. The blood-vessels in *all* organs, may, as to their disposition, be compared to the canals which are cut for the irrigation of a meadow;—they are carriers of nutritive matter—they surround, but do not themselves penetrate, the islands which they enclose; so that the only difference between the parts called *non-vascular* and the rest of the body is that which relates to the size of islets surrounded by the blood channels. The whole question thus resolves itself into one of degree, not of kind.

We close our remarks on nutrition by the following passage:—

"From the foregoing details the obvious inference results,—that each part of the organism has an individual Life of its own, whilst contributing to uphold the general Life of the entire being. This Life, or state of Vital Action, depends upon the due performance of the functions of all the subordinate parts, which are closely connected together. The lowest classes of organized beings are made up of repetitions of the same elements; and each part, therefore, can perform its functions in great degree independently of the rest. But, in ascending the scale, we find that the lives of the individual parts become gradually merged (so to speak) in the general life of the structure; for these parts gradually become more and more different in function, and therefore more and more dependent on each other for their means of support; so that the activity of all is necessary for the maintenance of any one. Hence the interruption of the function of any important organ is followed by the Death of the whole structure: because it interferes with the elaboration, circulation, or purification of that nutritious fluid, which supplies the pabulum for the growth and reproduction of the individual parts. But *their* lives may be prolonged for a greater or less duration, after the suspension of the regular series of their combined actions; hence it is, that *molecular* Death is not always an immediate sequence of *somatic* Death." P. 580.

In the chapter on Secretion many additional and important researches have been incorporated with the present edition; but our notice has already been so much extended, that we can only call attention to the admirable investigations of Mr. Bowman into the structure of the Malpighian bodies of the kidneys, and to those of Liebig and Lehmann respecting the chemical analysis of the urine.

The work concludes with a description of the reproductive process. This branch of physiology has received a vast addition in late years from the inquiries of such writers as Baer, Purkinje, Wagner, W. Jones, Coste, Valentin, and still more recently by the admirable researches of Martin Barry and Bischoff. Our space, however, will allow us merely to notice one or two of the latest facts which have been established.

One of the most novel of these additions relates to the maturation and discharge of ova in the human female at the menstrual period. It is a general law that the ova in all animals when they have attained their maturity, *whether impregnated or not*, are discharged from the ovary. The oviparous animals, for obvious reasons, afford the most striking illustration of this law, inasmuch as their large ova are readily recognised: now in these, as in the frog, tortoise, and common fowl, it is well known that eggs are laid even when the male has had no access to the female. But in the case of mammals, the minute size of the ovum renders similar observations upon their discharge a much more difficult matter; and hence it has happened, that although many physiologists, among whom we may mention Dr. Haighton and Dr. Blundell, have determined the fact that ova may become matured and be discharged independently of sexual connexion, yet these were regarded as occasional phenomena which could not be referred to any general law. Latterly, however, and especially by the careful observations of Dr. R. Lee, MM. Gendrin, Negrier, Raciborski, Bischoff, and Dr. Ritchie, it has been rendered very probable that at every menstrual period an ovum, already sufficiently developed to become, under the necessary conditions, impregnated, escapes from the ovary into the uterus. The inference therefore is, that a short time prior to menstruation, an ovum is prepared and ready for fecundation; that, consequent upon this preparation, a vascular activity is set up in the uterus, which, as it is well known, sympathizes in a remarkable and constant manner with the ovarium, in order to form the decidua; that, no impregnation occurring, the enlarged ovum bursts from its ovi-sac or Graafian follicle and descends by the Fallopian tube into the uterus, and forming no attachment to the inner surface of that organ, escapes externally; whilst the enlarged uterine blood-vessels, having now no functional office to accomplish, are relieved by the catamenia, and thus shortly return to their more ordinary condition. This is probably the usual process which appears to be analogous to the *heat* of the lower animals; but there are many deviations from it, so that "menstruation may take place without any such rupture; whilst, on the other hand, the maturation and discharge of mature ova may occur in the intervals of menstruation." (P. 688.)

The phenomena above noticed belong to a more general law of the animal economy, called by the German writers *Organic Periodicity*, and to which considerable attention has of late been directed in this country by Dr. Laycock, and by Quetelet and others on the Continent. That such a law does prevail is rendered probable by the diurnal variations in the frequency of the pulse observed by Dr. Guy; by similar variations in the quantity or urea secreted, this being most abundant in the morning, and diminishing in quantity towards the evening; but more especially by the phenomena of disease, such, for example, as the occurrence of tertian and quartan intermittent fevers. We are therefore inclined to agree with Valentin, who, in his recent work on physiology, remarks:—"our body presents not only a very high degree of symmetry in relation to its structure, but likewise so great an one in respect to time, that many, if not all the vital actions, appear to be subject to a definite periodicity."

An abstract of Dr. Ritchie's interesting researches respecting the exact nature of the corpus luteum is given. A variety of opinions have, it is well known, been entertained as to the seat of the deposit which occurs after the escape of the ovum, and which produces the corpus luteum; whether it be placed within the ruptured Graafian follicle, between its layers, or on its exterior. Dr. Ritchie contends that corpora lutea may be found in each of these situations, and moreover that their aspect and character may vary in a remarkable degree, some *(corpora albida)* being of a white colour, and either of a soft fatty aspect or dense and shining; others *(c. cephaloidea)* are yellowish and of a brain-like character; and another class *(c. rubra)*, which are at first similar to the last, although they are plumper, more vascular, better developed, and subsequently the granular matter of which they are composed, becomes of a decided red colour. Another, but less important class, is also described, consisting merely of the attenuated and ruptured follicle, without any particular matter being deposited. This inquiry is so important, that the following remarks of Dr. Carpenter will not be misplaced.

" The number of cases examined by Dr. Ritchie is not, perhaps, sufficient to enable us to found any positive statements upon the results of his examination of them; but the following inductions appear highly probable. 1. That the presence of *Corpora Rubra* may be regarded as indicative, not only of conception, but also of an advanced stage of pregnancy, or of recent delivery; but that their absence is not to be regarded as any proof to the contrary.—2. That the presence of *Corpora Cephaloidea* of the second order is to be regarded as indicative of conception.—3. That the presence of the *Corpora Cephaloidea* of the first order, or of *Corpora Albida*, cannot be regarded as in the least degree indicative of Conception; as they may result from the simple discharge of an Ovum, in the ordinary course of those changes to which the Ovarium is subject.—The excess of Corpora Albida above every other appearance is due, not merely to their being an ordinary result of the discharge of unimpregnated Ova; but also to the frequency of their production as degenerated forms (so to speak) of the Corpora Cephaloidea and Corpora Rubra of the gravid female; and also to their occasional existence as, from the first, the only Ovarian change following upon Conception." P. 693.

In his account of the ovum the author has omitted to notice that, as it traverses the fallopian tube, a considerable layer of albuminous matter is deposited on the exterior of the zona pellucida or vitelline membrane. This addition accounts for the increase which it has long been known the mammiferous ovum experiences, after it escapes from the ovary; but the observation of Professor Bischoff, for we are indebted to that admirable physiologist for ascertaining the fact, is of higher interest, because it tends to show the perfect uniformity which prevails in the formation and development of the ovum among the several classes of the vertebrata. It is known, for example, that the egg, as it passes through the oviduct of oviparous animals, that of the fowl for example, receives the white or albumen, and there can be no doubt that the deposit just alluded to is the analogous substance, derived from the highly organized mucous surface of the fallopian tube, or oviduct of the mammal.

A circumstance which has given rise both in this country and on the Continent to much speculation, relates to the proportional numbers of male and female births. It is well known that the former considerably exceed the latter, so that, " taking the average of the whole of Europe, the proportion is about 106 males to 100 females." There are, however, certain remarkable modifications of this law, and amongst others it has been observed, in illegitimate births, that in some places the males are to the female infants only as 102½ to 100. The cause of this difference has been variously explained. Dr. Carpenter thinks—

" This is probably to be accounted for by the fact, which is one of the most remarkable contributions that have yet been made by Statistics to Physiology, that the Sex of the offspring is influenced by the relative *ages* of the parents. The following table expresses the average results obtained by M. Hofacker in Germany, and by Mr. Sadler in Britain; between which it will be seen that there is a manifest correspondence, although both were drawn from a too limited series of observations. The numbers indicate the proportion of Male births to 100 Females, under the several conditions mentioned in the first column.

| Hofacker. | | Sadler. | |
|---|---|---|---|
| Father younger than Mother | 90·6 | Father younger than Mother | 86·5 |
| Father and Mother of equal age | 90·0 | Father and Mother of equal age | 94·8 |
| Father older by 1 to 6 years | 103·4 | Father older by 1 to 6 years | 103·7 |
| . . 6 to 9 | 124·7 | . . . . 6 to 11 | 126·7 |
| . . 9 to 18 | 143·7 | . . . . 11 to 16 | 147·7 |
| . . 18 and more | 200·0 | . . . . 16 and more | 163·2 |

From this it appears, that the more advanced age of the Male parent has a very decided influence in occasioning a preponderance in the number of Male infants; and, as the state of society generally involves a condition of this kind in regard to marriages, whilst in the case of illegitimate children the same does not hold good, the difference in the proportional number of male births is accounted for." P. 726.

There is, however, good reason to conclude that some other and probably more general causes are operative in this matter, for another great class of births, the *still-born*, present deviations from the usual law as marked as those just quoted. We have been favoured with some interesting observations upon this subject from a gentleman justly celebrated for the extent and accuracy of his statistical knowledge, Mr. Finlaison, the Government Actuary, from which we make the following selection. " I have for many years been of opinion, that the condition of the parents at the moment of conception has a decided influence upon the sex of the infant then called into existence. That the still-born are, in the vast majority of cases, the offspring of affliction is too evident to require illustration. It has long been known that they form $3\frac{1}{2}$ per cent. of the total births, so far as they are known; but if all were known the proportion would be close upon 5 per cent. Now there is no fact more constant than this, that, in this class, for every 100 girls there are 140 boys, notwithstanding that in France this proportion falls to 134." In another class, among whom it must be inferred there is deep distress, namely, in the women who are delivered in the Dublin Lying-in-Hospital, the number of boys considerably exceeds the usual average, and the same thing has been ascertained by Mr. Finlaison as regards one of the most suffering of the metropolitan districts, Bethnal-green. If we now turn " to the offspring of pampered wantonness," the contrast is great. It appears from the *Paris Annuaire*, that the number of disowned natural children amounted in five years to 17,409 boys and 17,117 girls, the sexes thus approaching to an equality. The deductions of Mr. Finlaison would indicate that, whenever the misery of a population, usually determined by the want of sufficient food, reaches a certain pitch, the number of females born, which must subsequently determine the progress of population diminishes, and thus the evil resulting from an excess of inhabitants in any country ultimately corrects itself.

We here conclude our notice of Dr. Carpenter's work, which we commend to the careful perusal of our readers, containing as it does a comprehensive, lucid, and philosophical exposition of the existing knowledge of this most interesting and important science.

